# Differences in Tetracycline Antibiotic Resistance Genes and Microbial Community Structure During Aerobic Composting and Anaerobic Digestion

**DOI:** 10.3389/fmicb.2020.583995

**Published:** 2020-10-16

**Authors:** Luyun Luo, Chengjia Zhang, Zhuo Zhang, Jing Peng, Yongqin Han, Pei Wang, Xiaoting Kong, Hamid Muhammad Rizwan, Deyong Zhang, Pin Su, Yong Liu

**Affiliations:** ^1^Yangtze Normal University, Chongqing, China; ^2^Hunan Plant Protection Institute, Hunan Academy of Agricultural Sciences, Changsha, China; ^3^College of Plant Protection, Hunan Agricultural University, Changsha, China

**Keywords:** manure, ARGs, aerobic composting, anaerobic digestion, microbial community

## Abstract

Antibiotics are widely added to swine forage and are the main reason for the environmental accumulation of antibiotic resistance genes (ARGs) in swine manure-dwelling microorganisms. Aerobic composting (AC) and anaerobic digestion (AD) are efficient methods for converting swine manure to bio-fertilizer while degrading residual antibiotics. However, the influence of these methods on ARG accumulation and the difference in their efficiency have rarely been investigated. In this study, we explored the variations in four tetracycline antibiotics (TCs) and their associated ARGs and in microbial communities after AC and AD treatment. After full-scale manure AC and AD, the four TCs were removed effectively. AD had a higher TC removal efficiency than AC and a slower rate of TC-associated ARG accumulation. In addition, the community structure was more stable in the AC and AD manures than in untreated manure, and the relationship among microbial species also evolved into competition from mutualism after both AC and AD treatment. It was also speculated that the genera *Acholeplasma* and *Arthrobacter* were the possible hosts of *tetO*, *tetW*, and *tetQ*; the shift in the prokaryotic community composition and the alleviation of selective pressure by TC degradation led to decreased relative abundance of ARGs in AD- and AC-treated manure.

## Introduction

Antibiotics have been heavily used for disease treatment in livestock and aquaculture, and in swine farming as feed additives (Zhang et al., 2015; Henriksson et al., 2018). The existence of antibiotic resistance genes (ARGs) has led to great concern due to their potential to generate highly antibiotic-resistant pathogens (Wright, 2010; Henriksson et al., 2018). According to a previous survey, China used a total of 162,000 tons of antibiotics in livestock farming in 2013 alone (Zhang et al., 2015; Sui et al., 2016), and animal manure has become a main source of the residual antibiotics and associated ARGs released into the environment (Zhao et al., 2010). Antibiotic residues in manure can enter the soil-water ecosystem, threatening the environment and human health (Ramaswamy et al., 2010; Awad et al., 2014).

Aerobic composting (AC) and anaerobic digestion (AD) are widely promoted practices for the management of animal waste because the residual antibiotics can be efficiently degraded by the abundant microorganisms that colonize the manure under AC or AD conditions (Ward et al., 2008; Diehl and Lapara, 2010; Selvam et al., 2012; Sun et al., 2016). However, the AC process often leads to a significant increase in ARGs because ARGs are highly mobile among the genomes of drug-resistant strains, and the accumulation of specific ARGs in certain bacterial genomes consequently causes the accumulation of ARGs for specific antibiotics (Ghosh and Lapara, 2007; Looft et al., 2012; Zhu et al., 2013; Su et al., 2015). These drug-resistant strains harboring specific ARGs are important sources of ARGs in the environment (Enne et al., 2001) when distributed into the environment (Li et al., 2015a). AD is recognized as an important approach for manure management that effectively degrades residual antibiotics and efficiently converts animal waste to green bio-fuel (Ward et al., 2008). Evidence has shown that thermophilic AD can also significantly reduce the accumulation of ARGs carried by animal manure (Diehl and Lapara, 2010; Ma et al., 2011); however, other studies have demonstrated that increased abundance of ARGs in agricultural soils was detected after AD-treated manure was applied as bio-fertilizer (Aydin et al., 2015; Cheng et al., 2016; Sui et al., 2016). At present, there is no corroborating evidence suggesting that AD should or should not be promoted in manure treatment with respect to the risk of environmental ARG contamination. Since both AC and AD are readily available approaches for animal manure management on an industrial scale, knowledge regarding their comparative performance in terms of antibiotic degradation and ARG accumulation is necessary to find a suitable method for manure management.

Microorganisms play a crucial role in the degradation of antibiotics and as hosts for ARGs during both AC and AD processes (Mitchell et al., 2013; Shi et al., 2016). Zhang et al. (2006) indicated that composting is a new alternative bioremediation technology for the treatment of antibiotics. Song et al. (2017) also demonstrated that bacterial communities accounted for 46.7% of the total variation in the overall ARG profiles during the AD process. In addition, the physicochemical properties during manure treatment also contribute to the efficiencies of antibiotic degradation by modulating microbial community structures. Song et al. (2017) indicated that these physicochemical properties [soluble chemical oxygen demand) SCOD, pH, NH_4_^+^, and volatile fatty acids (VFAs)] accounted for 30.8% of the total variation in the overall ARG profiles during the AD process. In this study, we used swine manure, which is rich in four tetracycline antibiotics (TCs), as a raw material to compare the performance of AC and AD processes in terms of antibiotic degradation efficiency and accumulation rate of TC-associated ARGs. Moreover, using 16S rRNA gene amplicon sequencing and qPCR analysis, we investigated the bacterial community composition and relative abundance of 11 TC-associated ARGs. The physicochemical properties and TC concentration dynamics under both conditions were also correlated with bacterial community analysis by using the Mantel test, canonical correspondence analysis (CCA), and CCA-based variation partitioning analysis (VPA). Our results provide a view of the comparative performance of AC and AD and will be valuable for designing a proper approach for livestock management.

## Materials and Methods

### Sample Collection

A full-scale pig manure AC and AD operation (mesophilic biogas plants) was performed from September to October 2017 in Shijiang village, Liuyang, Hunan, China. Raw swine manure was collected throughout the swine growth cycle from a neighboring swine manure disposal factory, where pigs were raised using TCs, including tetracycline (TC), oxytetracycline (OTC), chlortetracycline (CTC), and doxycycline (DOX). The pig manure was mixed with an automatic agitator. Fresh pig manure was divided into three parts: the raw manure, aerobic compost and AD samples. The six raw manure samples were collected before the aerobic compost and the AD operation sample. The compost manure was divided into six parts under a sunshade to shield the manure from sun and rain. Another portion of the manure was thrown into six mesophilic biogas plants (BGPs) for AD operation. The AD treatment samples were obtained directly using a 1.5 m long probe sample. After full-scale swine manure composting and AD operation (45 days), 0.2 kg samples were collected in September 2017. Eighteen composite samples were taken, each obtained by mixing three subsamples. The compost and digestate samples were transported to the laboratory and stored in a deep freezer at −20°C. All samples were vacuum freeze-dried and homogenized through a 100-mesh sieve for subsequent analysis.

### Extraction and Analysis of Antibiotics

The TCs detected in our study included four antibiotics: TC, OTC, CTC, and DOX. Simultaneous determination of veterinary antibiotics was performed in manure, compost, and sludge by liquid chromatography-tandem mass spectrometry by the isotope-labeled internal standard method as previously described (Huang et al., 2013; Pinheiro et al., 2013; Zhang et al., 2014). Briefly, extraction solutions were used to extract the selected antibiotics from pig manure, compost, and sludge samples. First, 1.0 g of fresh defrosted sample was placed into a 15 mL tube with 1 mL of methanol containing 1% acetic acid, and then, the sample was centrifuged at 23,074 × *g* for 30 s and then sonicated for 30 min in an SB-800D sonication bath (Ningbo, China). After centrifuging for 10 min at 2,185 × *g*, the supernatant was re-extracted twice as described. The extracts were made up to 3 mL with methanol containing 1% acetic acid and filtered through 0.22-μm syringe filters (Shanghai, China) for HPLC analysis.

### Physicochemical Properties

The pH of the manure samples was measured in the aqueous extract (soil: deionized water = 1:5) using a multi-parameter water quality-monitoring instrument. The levels of total nitrogen (TN, measured according to the modified Kjeldahl method, HJ/T 707-2014), total potassium (TK, measured according to flame atomic absorption spectrophotometric method, GB 9836-1998), total phosphorus (TP, measured according to the sodium hydrogen carbonate solution-Mo-Sb anti spectrophotometric method, HJ/T 704-2014), and organic matter (OM, measured according to the method for determination of soil organic matter, GB9834-1988) were measured by the Institute of Soil Science, Chinese Academy of Sciences (Nanjing, China).

### DNA Extraction, Amplification, and Quantitative Polymerase Chain Reaction (qPCR)

Eighteen swine manure, compost, and sludge samples were used for microbial community and TC-associated ARG analysis. Briefly, the MP FastDNA SPIN Kit for Soil (MP Biochemicals, Solon, OH, United States) was used to extract total genomic DNA following the manufacturer’s instructions. DNA was purified with the E.Z.N.A.^®^ Gel Extraction Kit, and the final amplicon was quantified using a Qubit^TM^ 2 Fluorometer. The extracted DNA was diluted to a concentration of 30 ng/μL and used as a template for PCR amplification. The universal primers 343F (5′-TACGGRAGGCAGCAG-3′) and 798R (5′-AGGGTATCTAATCCT-3′) were used to amplify the V3-V4 variable regions of the 16S rRNA genes (Lin et al., 2017).

The total community DNA was used as the template to amplify 11 TC-associated ARGs and total bacterial 16S rRNA gene fragments. These ARGs were selected because they are widely detected in livestock farms worldwide (Pruden et al., 2006; Wu et al., 2010; Cummings et al., 2011; Zhu et al., 2013). Eleven TC-associated ARGs were detected by a single PCR. Then, qPCR was used to quantify the abundance of the detected target ARGs. The relative abundance of the ARGs was determined by the ARG/16S rRNA gene copy numbers. The qPCR primers (Aminov et al., 2001, 2002; Ng et al., 2001) and annealing temperatures of each target gene are listed in Supplementary Table S1. The qPCR mix for bacteria (20 μL) contained 10 μL of 2 × AceQ qPCR SYBR qPCR Master Mix, 0.4 μL of forward and reverse primers (10 μM), 8.6 μL of nuclease-free water, and 0.6 μL of genomic DNA as a template. The PCR program used was as follows: activation at 95°C for 10 min and 40 cycles of denaturation for 45 s at 95°C, annealing for 45 s as described in Supplementary Table S1, and extension for 30 s at 72°C, followed by melting curve analysis from 65 to 95°C. Triplicate reactions were performed for each sample, standard, and negative control. Standard curves were obtained using quantified 10-fold serial dilutions of target plasmid DNA with a CFX96TM real-time PCR system (Bio-Rad, China) using AceQ qPCR SYBR Green Master Mix (Vazyme, China).

### 16S rRNA Gene Sequencing of Bacterial Communities

The mixed PCR products were sequenced (2 × 250 bp) on an Illumina MiSeq platform by ANNOROAD Gene Technology Co., Ltd. (Beijing, China) according to standard protocols. Raw sequence data reads were processed with a series of bioinformatics tools. In brief, a separate sample was generated according to different 12 bp barcodes and primers, allowing for one mismatch. The Trimmomatic (Bolger et al., 2014) and FLASH (Reyon et al., 2012) software programs were used to assemble and filter the raw fastq data and to remove the low-quality sequences with average quality scores less than 20 and ambiguous bases (N). The assembled sequences were divided into separate samples with different barcodes. The data were subsequently analyzed using QIIME software (version 1.8.0) (Caporaso et al., 2010). Reads in which 75% of the bases had average quality scores above 20 were retained; the chimera was detected and removed. USEARCH software was used to cluster all sequences with 97% similarity cutoff to generate operational taxonomic units (OTUs) (Edgar, 2013). The RDP Classifier database (Silva database 132 version) with a 70% confidence threshold was used to assign the representative microbial sequences for each OTU (Wang et al., 2007). The re-sampled OTU table was obtained randomly with the lowest number of sequences (14,313) for bacteria and used for the subsequent analysis.

Alpha diversity indices, including the Shannon, InvSimpson, observed richness, and Chao1 indices, were calculated in this study. Principal coordinate analysis (PCoA) was used to demonstrate the microbial community differences among the three groups (Caporaso et al., 2010). To determine the differences among the bacterial communities in these three groups, three non-parametric tests (MRPP, Adonis, and ANOSIM) were used in this study (Anderson, 2010). The Mantel test, canonical correlation analyses (CCA), and CCA-based VPA were used to measure the variations in different environmental factors. In this study, all the raw bacterial sequences were deposited in the SRA database (SRR8471879-SRR8471894).

### Network Construction and Analysis

The phylogenetic molecular ecological networks (pMENs) of the three groups (PM, AC, and AD) were constructed using a molecular ecological network analysis pipeline based on the Spearman rank correlation matrix (MENA)^1^ (Zhou et al., 2010, 2011; Deng et al., 2012). The peripherals were those OTUs that had links staying within their respective modules. Generalists included module hubs (nodes that were highly connected with nodes within their modules, *Zi* > 2.5) and connectors (nodes that were connected with several modules, *Pi* > 0.62). The network of the population interaction plot was visualized with Cytoscape 3.6.0 software. Spearman correlation analysis was performed between TC RGs and the top 30 genera.

### Statistical Analysis

The difference in relative abundance among the top 30 genera was assessed using STAMP software v2.1.3. The physicochemical properties, TC content and alpha diversity indices were analyzed by performing a one-way ANOVA followed by Duncan’s multiple-range test using SPSS 21.0 software (IBM Co., Armonk, NY, United States). Correlation analysis among bacterial populations, physicochemical properties, and ARGs was also performed with the Spearman method using SPSS 21.0 software (IBM Co., Armonk, NY, United States).

## Results

### Dynamics of Antibiotic Residuals During AC and AD Processes

**FIGURE 1:**
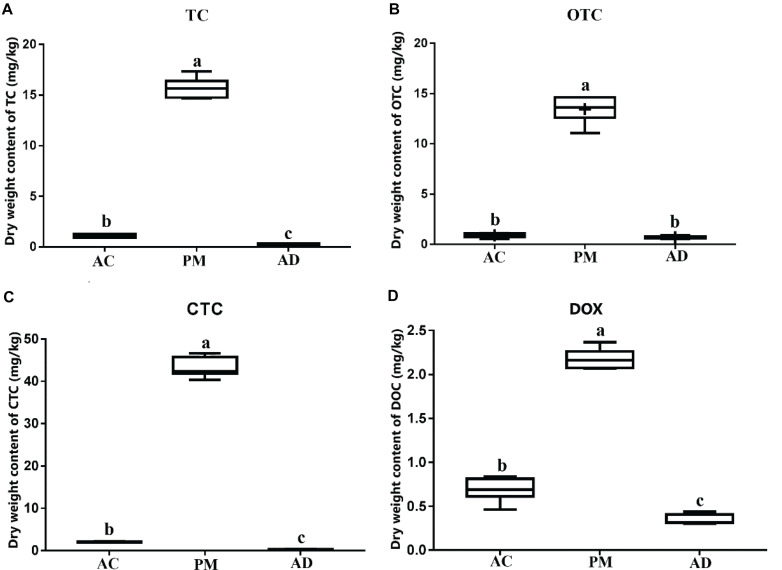
**(A)** Dry wight content of tetracycline (TC). **(B)** Dry wight content of oxytetracycline (OTC). **(C)** Dry wight content of chlortetracycline (CTC). **(D)** Dry wight content of doxycycline (DOX). Dynamics of TCs antibiotics (OTC, TC, CTC, and DOX). PM, swine manure samples; AC, aerobic composting samples; AD, anaerobic digestion samples. The error bar indicates standard error.

A significant impact was observed on the residues of all antibiotics tested during manure AC and AD. The levels of all four TC antibiotics decreased over time (Figure 1). The TC, OTC, and CTC residual amounts were less than 10% in the AC and AD treatment samples. In addition, the DOX residual amount was 31.79% in the AC group and 15.91% in the AD group. There was no significant difference in the level of OTC antibiotics between the AC and AD treatment samples. The levels of antibiotics (TC, CTC, and DOX) in the AC treatment samples was significantly higher than that in the AD treatment samples (*p* < 0.05) (Figure 1).

### Physicochemical Properties

The physicochemical properties of the samples from the three groups are summarized in Supplementary Table S2. There was a significant difference among these physicochemical properties, including differences in pH, OM, and TN. Among these physicochemical properties, pH and OM were the highest in the raw manure samples and lowest in the AD treatment samples (Supplementary Table S2). The TN content was highest value in the AD treatment samples and lowest value in the treated manure samples. There was no significant difference in TP and TK content among the three groups.

### Composition of ARGs During AC and AD Processes

**FIGURE 2:**
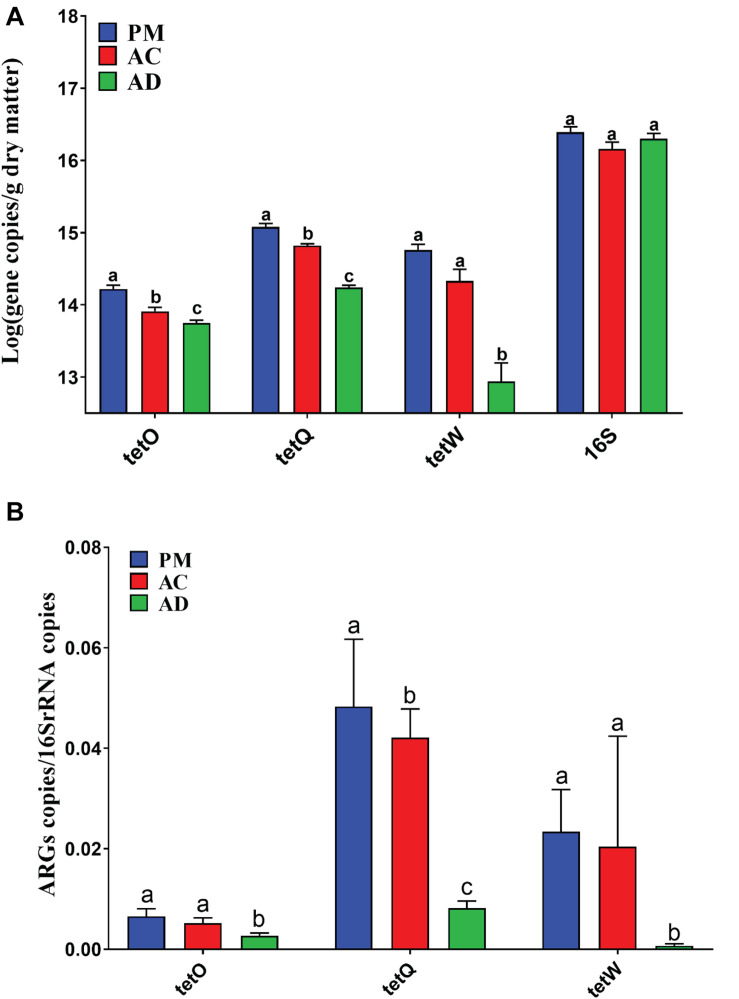
Lg-transformed of the ARGs or bacterial 16S rRNA gene copies **(A)** and relative abundance of ARGs among three group samples **(B)**. PM, swine manure samples; AC, aerobic composting samples; AD, anaerobic digestion samples. The difference in these ARGs or 16S rRNA gene copies, and relative abundance of ARGs among three group samples was assessed by performing a one-way ANOVA followed by Duncan’s multiple range test (*p* < 0.05). The error bar indicates standard error.

Three of the 11 TC-associated ARGs were detected in the untreated swine manure samples. The qPCR results showed that there were significant differences among samples from all three groups (Student’s *t*-test, *p* < 0.05) (Figure 2). In addition, the relative abundance of ARGs (*tetO*, *tetW*, and *tetQ*) was significantly higher in samples from the raw manure group than the AC and AD groups (Figure 2). The total relative abundance of ARGs was also significantly higher in the raw manure group than in the AC and AD groups (*p* < 0.05). The total relative abundance of ARGs decreased by 85.26% in the AD treatment samples but only by 13.45% in the AC treatment samples. Spearman correlation analysis showed that there was a significant positive correlation among three TC-associated ARGs (*tetO*, *tetW*, and *tetQ*) (Supplementary Table S3). The relative abundance of these ARGs was also significantly positively correlated with TC concentration.

### Response of Bacterial Communities During AC and AD Processes

A total of 453,666 high-quality 16S rRNA gene sequences were obtained from 18 samples by Illumina MiSeq sequencing, ranging from 15,110 to 41,541 reads per sample. Then, 2,321 OTUs were generated after re-sampling with the minimum sequences. *Firmicutes* (42.46%), *Bacteroidetes* (28.86%), *Proteobacteria* (19.79%), and *Actinobacteria* (3.88%) accounted for 95% of the bacterial sequences. The bacterial communities of the raw manure samples were significantly different from those of the AC and AD treatment samples (Table 1). *Actinobacteria* (3.54–10.32%), *Bacteroidetes* (3.24–21.26%), *Firmicutes* (31.09–67.41%), and *Proteobacteria* (14.15–40.85%) were the dominant phyla in the AC treatment. However, the bacterial communities in the AD group samples were dominated by *Bacteroidetes* (40.89–53.78%), *Firmicutes* (28.27–44.55%), *Proteobacteria* (7.18–10.67%), *Cloacimonetes* (2.10–5.78%), and *Spirochaetae* (1.41–3.33%). The relative abundance of *Actinobacteria* and *Proteobacteria* was significantly higher (*p* < 0.05) in the AC treatment than in the AD group (Table 1). In addition, the relative abundance of *Bacteroidetes*, *Cloacimonetes*, and *Spirochaetae* was significantly higher (*p* < 0.05) in the AD group than in the AC treatment. *Cloacimonetes* were observed only in the AD treatment.

**TABLE 1:** The dominant phyla of three group samples.

**Phylum**	**AC**	**AD**	**PM**
Firmicutes	9.79 ± 3.75b	38.27 ± 3.14a	47.74 ± 6.55a
Bacteroidetes	29.79 ± 3.64b	44.42 ± 2.67a	11.59 ± 2.46c
Proteobacteria	39.95 ± 2.38a	8.92 ± 0.54c	31.05 ± 4.3b
Actinobacteria	16.71 ± 4a	0.73 ± 0.18b	6.91 ± 1.09b
Cloacimonetes	0.02 ± 0.02b	3.96 ± 0.64a	0 ± 0b
Spirochaetae	0.76 ± 0.27b	2.33 ± 0.27a	0.45 ± 0.12b
Tenericutes	0.23 ± 0.07a	0.33 ± 0.03a	1.82 ± 0.88a
Gemmatimonadetes	1.65 ± 0.17a	0.02 ± 0.01b	0.17 ± 0.07b
Fibrobacteres	0.56 ± 0.16a	0.31 ± 0.02a	0.01 ± 0b

*The difference in main phyla among three group samples was assessed by performing a one-way ANOVA followed by Duncan’s multiple range test (p < 0.05). PM, pig manure samples; AC, AC process group samples; AD, AD process group samples. The letter a, b, and c represent significant differences among groups, respectively.*

**FIGURE 3:**
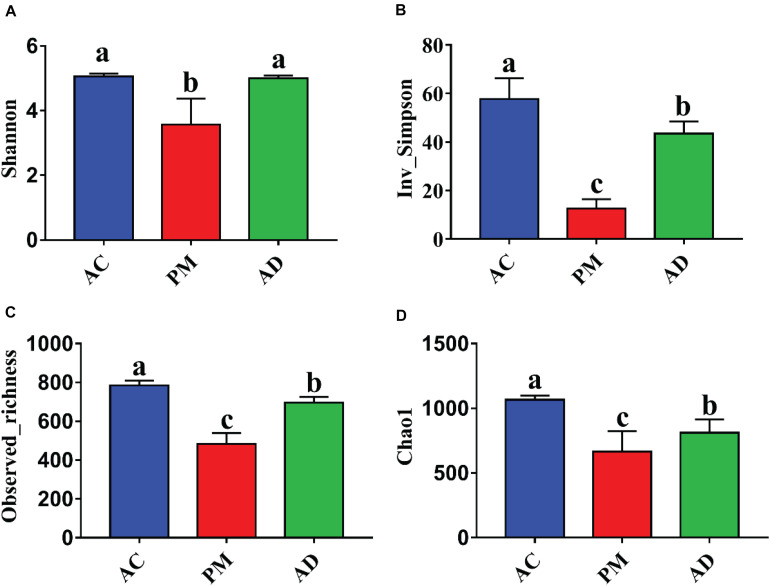
**(A)** The shannon index. **(B)** The Inv_Simpson index. **(C)** The observed_richness index. **(D)** The Chao index. Summary of α diversity indices. PM, swine manure samples; AC, aerobic composting samples; AD, anaerobic digestion samples. The error bar indicates standard error.

**FIGURE 4:**
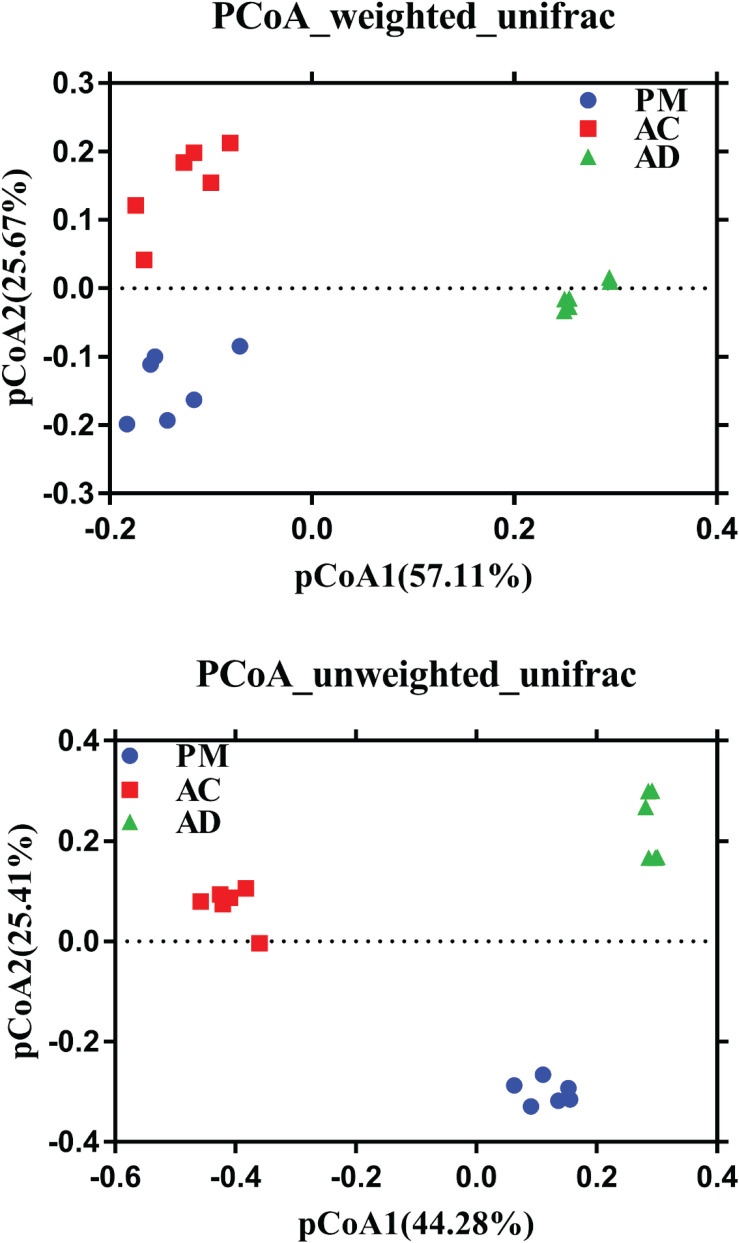
Principal coordinate analysis (PCoA) using weighted and unweighted UniFrac community distances between among three group samples. PM, swine manure samples; AC, aerobic composting samples; AD, anaerobic digestion samples.

The Shannon indices of the AC and AD treatments were significantly higher than those of the untreated manure group (Student’s *t*-test, *p* < 0.05, Figure 3). At the same time, higher microbial diversity and richness were observed in the AC and AD treatments than in the untreated swine manure group. The sample groups exhibited clear differences (*p* < 0.01) from one another and were further verified by non-parametric multivariate statistical tests of dissimilarity (MRPP, ANOSIM, and PERANOVA) based on Bray-Curtis distance and Jaccard distance among three groups (Table 2). The PCoA showed that all three (AC, AD, and PM) are clearly separated (Figure 4). The first coordinate (pCoA1) showed a 57.11% difference in community variation and separated the AD treatment samples and the other samples, and pCoA2 accounted for 25.67% of the dissimilarity (Figure 4).

**TABLE 2:** The dissimilarity analysis based on Bray-Curtis distance and Jaccard distance in the OTU level.

		**MRPP**		**ANOSIM**		**PERMANOVA**	
**Distance**							
**algorithm**	**Treatments**	**δ**	***p***	***R***	***p***	***F***	***p***
Bray_Curtis	PM-AD	0.4205	0.002	1	0.002	21.0829	0.003
	PM-AC	0.6136	0.002	1	0.002	8.7272	0.003
	AC-AD	0.5047	0.003	1	0.003	13.3217	0.002
Jaccard	PM-AD	0.4403	0.003	1	0.001	11.9585	0.002
	PM-AC	0.5535	0.001	1	0.003	10.0293	0.003
	AC-AD	0.4398	0.003	1	0.002	18.7414	0.003

*PM, untreated swine manure samples; AC, aerobic composting samples; AD, anaerobic digestion samples. The values of lower triangular matrices for MRPP, ANOSIM, and PERMANOVA are delta, R value and F-value, respectively.*

### Relationships Among Physicochemical Properties and Bacterial Communities

**FIGURE 5:**
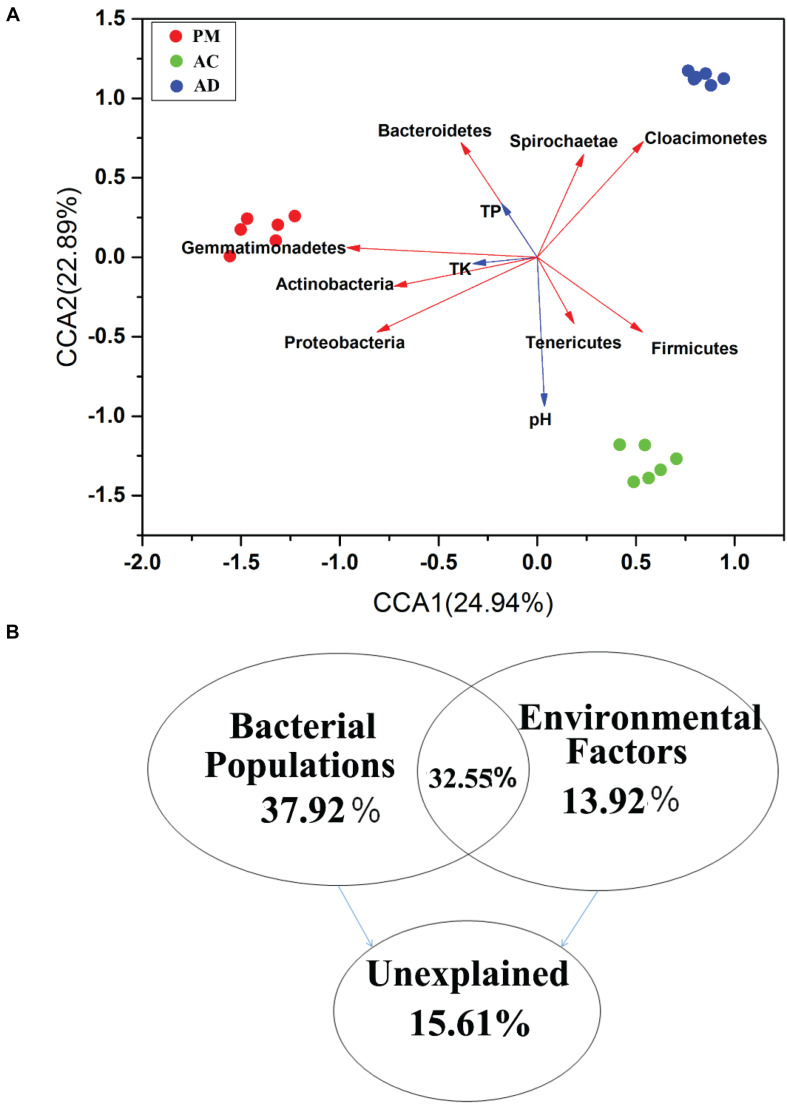
Canonical correspondence analysis (CCA) based on bray curtis distance **(A)** and CCA-based variation partitioning analysis (VPA) of bacterial communities explained by dominant bacterial populations **(B)**.

CCA was used to investigate the relationships between bacterial communities and physicochemical properties. Three clusters of bacterial communities were differentiated by CCA (Figure 5A). CCA1 and CCA2 accounted for 47.83% of the total variation in these bacterial communities (Figure 5A). The results of VPA showed that the dominant bacterial populations and five selected physicochemical properties (pH, OM, TP, TN, and TK) accounted for 37.92% and 13.92% of the total variations during the AC and AD processes, respectively (Figure 5B). Moreover, the variation partition analysis (VPA) also accounted for 32.55% of the total variations. Only 15.61% of the total variations were shown by other factors. Among these dominant bacterial populations, *Gemmatimonadetes* and *Proteobacteria* shared a strong positive correlation with bacterial communities (Mantel: *r* > 0.5 and *p* = 0.001) based on the Bray-Curtis distance and Jaccard distance matrix (Supplementary Table S4). The pH, OM, TN, and TC (TC, OTC, CTC, and DOX) concentrations were strongly positively correlated with bacterial communities (Mantel: *r* > 0.5 and *p* = 0.001) based on the Bray-Curtis distance and Jaccard distance matrix.

### Molecular Ecological Networks During AC and AD Processes

**FIGURE 6:**
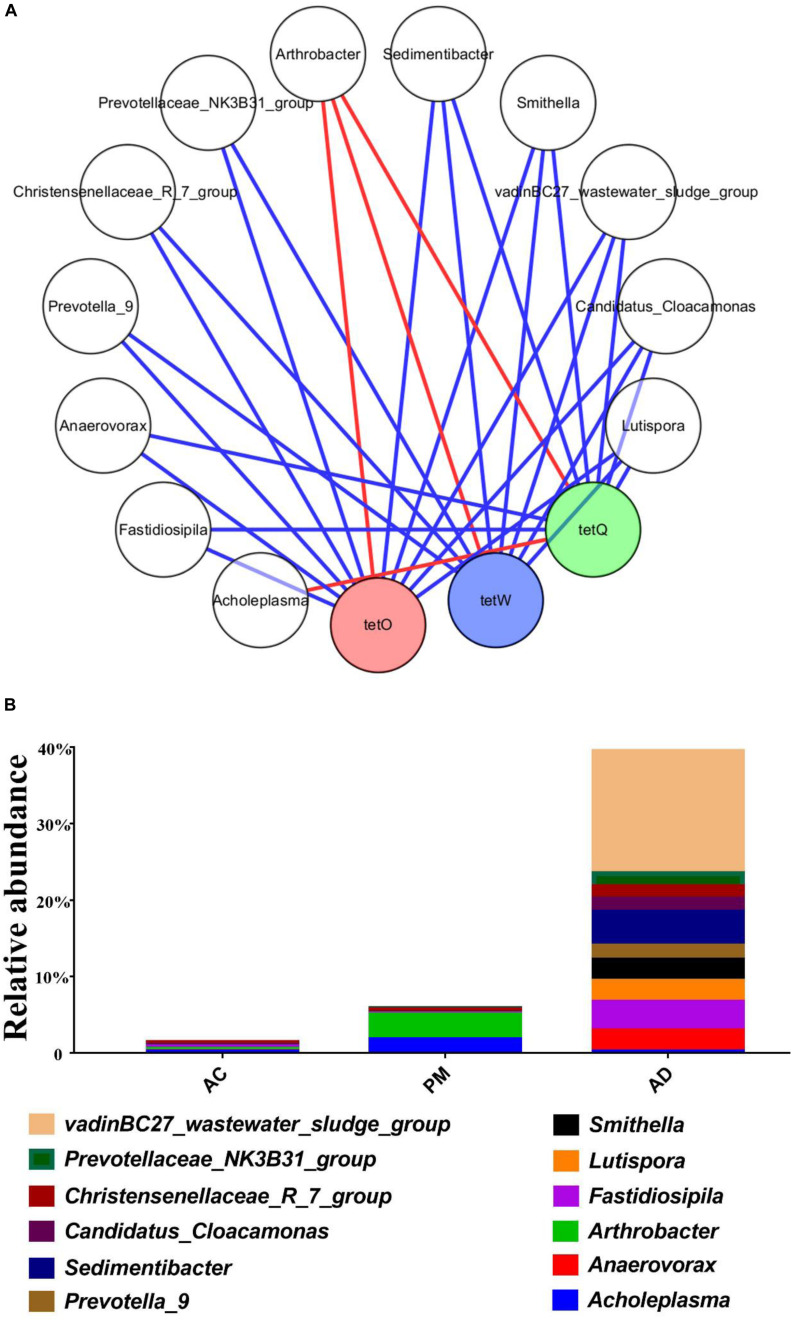
Network analysis of the co-occurrence patterns between ARGs and microbial taxa **(A)** and abundance of these bacterial genus **(B)**. A connection represents a significant correlation (|R| > 0.6 and *p* < 0.05; Red: positive, Blue: negative).

The pMEN values attributed to the bacterial communities are summarized in Table 3. The AD treatment (avgK, 13.828) and AC treatment (avgK, 12.055) had higher average connectivity than the untreated swine manure group (avgK, 6.67). The nodes and links for untreated swine manure (224, 747), AC treatment (361, 2,176), and AD treatment (511, 3,533) were also obtained in this study (Table 3). At the same time, the three groups also had higher modularity values than the random networks. The overall pMENs of these three groups are shown in Supplementary Figures S3, S4. A total of 5, 6, and 8 modules were obtained for the untreated swine manure, AC treatment, and AD treatment, respectively. A majority of the nodes in the three networks belonged to the phyla *Actinobacteria* (0.55–13.92%), *Bacteroidetes* (9.22–32.31%), *Firmicutes* (3.26–43.84%), and *Proteobacteria* (8.27–29.23%). Most of the positive interactions (57.91–86.61%) among the OTUs were observed in these three networks. The number of positive interactions was significantly lower in the AC and AD treatment samples than in the untreated swine manure samples. A majority of the OTUs (95.31%) were peripherals, and 4.69% of the bacterial OTUs were generalists (1.96% of the OTUs were module hubs, and 2.73% of the OTUs were connectors). In addition, most of the connectors belonged to the AC and AD treatments; only OTU_1589 (*Acidimicrobiales*) belonged to the untreated swine manure group, and the module hubs were also found in three groups. The genera *Acholeplasma* and *Arthrobacter* were positively (|R| > 0.6 and *p* < 0.05) correlated with the abundance of *tetO*, *tetW*, and *tetQ* and are shown in Figure 6. There was no obvious difference between the AC and AD processes (Supplementary Figure S2C). The other 10 genera were negatively (|R| > 0.6 and *p* < 0.05) correlated with the abundance of ARGs, as shown in Figure 6. Among these genera, the abundance of most genera (excluding *Christensenellaceae_R_7_group*, *Candidatus_Cloacamonas*, and *Lutispora*) was significantly higher in the AD treatment group than in the AC treatment group.

**TABLE 3:** The properties of the empirical and random networks under different treatments.

	**Empirical network**								**Random networks (100)**		
**Treatments**	**Modularity (fast_greedy)**	**Average path distance (GD)**	**Average clustering coefficient (avgCC)**	**Average degree (avgK)**	**Modularity number**	**Links**	**Nodes**	**RMT threshold**	**Modularity (fast_greedy)**	**Average path distance (GD)**	**Average clustering coefficient (avgCC)**
PM	0.518	6.481	0.365	6.67	17	747	224	0.91	0.317 ± 0.006	2.939 ± 0.038	0.109 ± 0.011
AC	0.551	5.008	0.446	12.055	12	2176	361	0.91	0.221 ± 0.004	2.755 ± 0.017	0.102 ± 0.007
AD	0.573	4.436	0.439	13.828	13	3533	511	0.91	0.216 ± 0.004	2.740 ± 0.010	0.060 ± 0.004

*PM, untreated swine manure samples; AC, aerobic composting samples; AD, anaerobic digestion samples.*

## Discussion

Previous studies have shown that AC and AD treatment can effectively degrade TC residues and reduce TC-associated ARGs in swine manure (Ward et al., 2008; Diehl and Lapara, 2010; Selvam et al., 2012; Sun et al., 2016). In this study, the dynamic changes in TCs and their associated ARGs, as well as the bacterial communities, were investigated after AC and AD processes. The AD treatment demonstrated superior TC degradation efficiency, as evidenced by the significantly low TC concentrations in AD-treated manure. Although previous studies have investigated the variations in ARGs during AC or AD processes (Diehl and Lapara, 2010), few studies have compared the differences in ARG abundance after AC and AD treatment. Our study also demonstrated that the relative abundance of three TC-associated ARGs (*tetO*, *tetQ*, and *tetW*) was decreased significantly after AC and AD treatment (Figure 2). The genes *tetO*, *tetQ*, and *tetW* were frequently detected with high abundance in samples from sewage treatment facilities, animal production waste, aquaculture areas, and untreated sewage (Zhang et al., 2009). The AD treatment showed significantly lower accumulation of the three TC-associated ARGs than the AC treatment (Figure 2). Correlation analysis revealed that the genes *tetO*, *tetW*, and *tetQ* were positively correlated with others (Table 2), indicating the possibly shared bacterial host or ecological function of these three genes (Li et al., 2015b). ARG-harboring bacteria are abundant in animal manure-supplemented agricultural soils (Heuer et al., 2011). Our results revealed that AD treatment could more effectively reduce ARG accumulation in comparison with AC treatment. This might contribute to prolonged effectiveness of antibiotics used to treat humans, especially when these ARG fragments are transferred to other potential pathogens through the food chain (Heuer et al., 2009; Muziasari et al., 2014; Singh et al., 2018).

ARGs can be located in various mobile genetic elements (MGEs), such as plasmids, integrons, transposons, insertion sequences, and other integrative conjugative elements, which are crucial tools for ARG dissemination among bacterial cells (Luo et al., 2010; Stokes and Gillings, 2011). In our study, the α-diversity of the bacterial community was found to be significantly higher in both AC- and AD-treated manure than in untreated manure (Figure 3). This was possibly because the AC and AD treatments created conditions that allowed more diverse microorganisms to grow. In this study, the microbial community in swine manure exhibited substantial shifts after the AC and AD treatments (Figure 4). The results of the dissimilarity test and PCoA indicated that the three different groups were clearly separated. The dynamic change in *tetO*, *tetW*, and *tetQ* abundance was strongly correlated with the bacterial community structure. We speculate that the differences in bacterial community structure between the AC and AD treatments, which are characterized by different physiochemical properties, resulted in the different accumulation rates of these three TC-associated ARGs. Importantly, the contamination of digestates with residues of antibiotics, metal compounds and detergents will determine whether MGEs carrying RGs might represent a selective advantage for their bacterial hosts (Wolters et al., 2016). The identified changes in the present study indicated that the selective pressure exerted by TCs was one of the factors leading to the decrease in the relative abundance of ARGs in the AC and AD processes.

Previous studies demonstrated that Firmicutes, Proteobacteria, and Bacteroidetes are the most dominant phyla in swine manure (Zhang et al., 2014). Firmicutes and Actinobacteria were the groups most likely to carry and transfer ARGs according to the study of Huerta et al. (2013). In this study, the CCA results showed that the abundance of Gemmatimonadetes, Actinobacteria, Proteobacteria, and Firmicutes was positively correlated with the abundance of ARGs (*tetO*, *tetQ*, and *tetW*) during the AC and AD processes (Supplementary Table S3). Song et al. (2017) suggested that Bacteroidetes and Spirochetes are responsible for the variation in TC-associated ARGs (*tetC, tetW, and tetQ*) after the AD process. In our study, CCA1 and CCA2 accounted for 47.83% of the total variation in the bacterial communities (Figure 5A). The results of VPA showed that the dominant bacterial populations accounted for 37.92% of the total variation in the overall TC-associated ARGs (Figure 5B). Tetracycline drugs are broad-spectrum antibiotics that inhibit the binding of aminoacyl tRNA to the ribosome binding site (A) to prevent the synthesis of bacterial proteins. The ribosome protectors are encoded by the *tetO*, *tetW*, and *tetQ* gene. Binding of the ribosome protectors to the ribosome causes a change in ribosome configuration, but does not alter or prevent protein synthesis (Ray et al., 1988; Taylor and Chau, 1996). The ribosomal protective protein are encoded by the *tetO* has a ribosome-dependent GTP hydrolase activity that, in the presence of GTP, weakens the binding capacity of tetracycline to the ribosome (Trieber et al., 1998). The amino acid homology of the *tetO* and *tetW* is similar. The *tetQ* gene is primarily associated with binding chromosomal elements and encodes their translocation, which allows plasmids to be transferred into other genera (Jones et al., 1992). Bacterial population play an important role in the evolution of ARGs (Song et al., 2017; Su et al., 2018). It is likely that the shifts in the relative abundance of specific ARGs and selective pressure reflected changes in the bacterial community composition under mesophilic conditions. In addition, the physicochemical properties also played an important role in the variation in the ARGs (Supplementary Table S4).

There was a significantly higher abundance of Bacteroidetes and Clostridia in the AC and AD treatment than PM treatment (Supplementary Figure S1). Wolters et al. (2018) found that OTUs assigned to Clostridia were significantly enriched, which might indicate that Clostridium representatives are potential *tetW* hosts in the maize plant rhizosphere. Clostridia typically have a high relative abundance in digestate bacterial communities (Schlüter et al., 2008; Wolters et al., 2016). Clostridia also were observed higher in relative abundance in municipal sewage sludge soil compared to control soil, with higher abundance of *tetW* (Wolters et al., 2019). Wolters et al. (2018) also found same trend for the digestate application, with direct enrichment of Clostridiales and Bacteroidetes. At the phylum level, Wolters et al. (2016) also found that relative abundances observed for Proteobacteria were lower in digestate samples compared to manure, same as our results. It might represent a risk deserving more attention in the next research. Drug-resistant bacteria play the most important role in the microbial degradation of antibiotics. However, the single strains isolated from the environment often have a good degradation effect, while the degradation ability is generally reduced when added to the complex environment such as soil or water. The reason may be that oxygen, pH, temperature, water and other factors between AC and AD process may also affect the growth and metabolism of the strain, thus affecting its ability to degrade antibiotics. AD process had a more efficient TCs degradation, which might be root cause for their ARGs difference.

In this study, ARG abundance was observed to be significantly correlated with physicochemical properties (pH, OM, and TN), which is consistent with a previous study conducted by Zhang et al. (2014). Compared to the untreated manure, the AC and AD treatments exhibited significantly reduced OM content and pH, but the AD treatment exhibited a higher OM degradation rate than the AC treatment (Supplementary Table S2). pH is considered a major factor determining soil bacterial diversity and composition (Song et al., 2017). Microbial degradation of OM may be the main reason for the decrease in the OM content and pH in all treatment samples. Thus, both physicochemical properties and the dominant bacterial populations are key determinants shaping bacterial community structures.

The results showed that most of the microbes had a limited number of connections, while few microorganisms were highly connected within the community. The higher modularity index in the corresponding randomized networks suggests that the network had a modular structure (Feng et al., 2017). Meanwhile, a large number of nodes and links were observed in the networks of the treatment samples (Table 3). This indicates a highly complex network, which usually indicates a highly stable community structure (Mougi and Kondoh, 2012; Liang et al., 2016). The observed co-occurrence network patterns indicated that the overall bacterial communities were affected by the AC and AD treatments. As described previously, mutualism and competition usually result in positive and negative interactions among populations (Deng et al., 2016). In this study, although most of the interactions between the OTUs were positive (57.91–86.61%), the observed positive interactions were significantly lower in the AC and AD treatment samples than in the swine manure group. This indicated that mutualism between populations evolved into competition due to adventitious microbial growth.

In addition, a Spearman correlation analysis between the TC-associated ARGs and microbial taxa was performed to identify the potential hosts of these ARGs (Li et al., 2015a; Figure 4). Zhang et al. (2009) also reported that *Bacteroides* species might be the hosts of *tetQ*. In the present study, *Acholeplasma* and *Arthrobacter* were observed to be potential host bacteria, which were positively (|R| > 0.6 and *p* < 0.05) correlated with the abundance of *tetO*, *tetW*, and *tetQ* (Figure 6). In addition, Huang et al. (2016) found that *Prevotella* was negatively correlated with *tetQ*, which is consistent with our results. Thus, the reduced abundance of TC-associated ARGs (*tetO*, *tetQ*, and *tetW)* was likely due to the decrease in the relative abundance of *Acholeplasma* and *Arthrobacter*. The significant positive correlations between these ARGs and bacterial populations indicated that these ARGs have the same host bacteria. However, there was no obvious difference between the AC and AD processes among the positive ARG carriers (Supplementary Figure S2C). Interestingly, genera (except *Christensenellaceae_R_7_group*, *Candidatus_Cloacamonas*, and *Lutispora*) for which relative abundance was negatively correlated with ARG abundance were present at significantly higher levels in the AD-treated samples than in the AC-treated samples. This phenomenon could explain the lower ARG abundance in AD-treated samples than in AC-treated samples. Nevertheless, further investigation is necessary to ascertain the mechanisms underlying the relatively low ARG accumulation rate in the AD process. Degradation of TC and different bacterial community composition might be the two main factors.

## Data Availability Statement

Publicly available datasets were analyzed in this study. This data can be found here: the SRA database (SRR8471879–SRR8471894).

## Author Contributions

LL, ZZ, and YH performed the experiments and analyzed the data. PW prepared the figures and tables. JP performed the experiments, analyzed the data, and authored or reviewed drafts of the manuscript. PW and XK performed the experiments. DZ and PS analyzed the data, contributed reagents, materials, and analysis tools, and authored or reviewed drafts of the manuscript. YL conceived and designed the experiments, analyzed the data, authored or reviewed drafts of the manuscript, and approved the final draft. HR conceived and designed the experiments, analyzed the data, contributed reagents, materials, and analysis tools, authored or reviewed drafts of the manuscript, and approved the final draft. All authors contributed to the article and approved the submitted version.

## Conflict of Interest

The authors declare that the research was conducted in the absence of any commercial or financial relationships that could be construed as a potential conflict of interest.

## Footnotes

1 http://ieg4.rccc.ou.edu/mena/login.cgi

## Supplementary Material

Supplementary Figure 1 Relative abundance of bacterial class among three group samples. PM, swine manure samples; AC, aerobic composting samples; AD, anaerobic digestion samples.

Supplementary Figure 2 Comparison of taxonomic distribution of major genera with significant differences (relative abundance of top 30 genus) between two group samples. PM, swine manure samples; AC, aerobic composting samples; AD, anaerobic digestion samples.

Supplementary Figure 3 Phylogenetic molecular ecological networks (pMENs) of bacterial communities at the PM **(A)**, AC **(B)**, and AD **(C)** groups. Modules with >5 nodes were obtained for three group (PM, AC, and AD), respectively. The links between two nodes show the correlation (red: positive, blue: negative). The size of cycle indicates the relative abundance of the OTU. The number in the center of circle represents the modules to which these OTUs belongs. PM, swine manure samples; AC, aerobic composting samples; AD, anaerobic digestion samples.

Supplementary Figure 4 Summary of module hubs and connectors of the bacterial communities of three groups (PM, AC, and AD). The OTUs were peripherals whose links mainly stayed within their respective modules. Generalists including module hubs (nodes that highly connected with nodes within their modules, Zi > 2.5) and connectors (nodes that connected with several modules, Pi > 0.62). PM, swine manure samples; AC, aerobic composting samples; AD, anaerobic digestion samples.

Supplementary Table 1 The qPCR primers used in this study.

Supplementary Table 2 Physicochemical properties of three group samples. The difference in physicochemical properties among three group samples was assessed by performing a one-way ANOVA followed by Duncan’s multiple range test (*p* < 0.05). PM, untreated swine manure samples; AC, aerobic composting samples; AD, anaerobic digestion samples.

Supplementary Table 3 Spearman’s correlation coefficients between relative abudance of ARGs gene, physicochemical properties, the dominant bacterial populations, and TCs concentration. ^∗^ indicate significant value at *p* < 0.05. ^∗∗^ indicate significant value at *p* < 0.01.

Supplementary Table 4 Mantel analysis on the relationship between the relative abundance of OTUs and environmental factors. *P*-values were calculated using the distribution of the Mantel test statistics estimated from 9,999 permutations.
