# Supplementary material for: Differences in Tetracycline Antibiotic Resistance Genes and Microbial Community Structure During Aerobic Composting and Anaerobic Digestion
Source: Front Microbiol. 2020 Oct 16;11:583995. doi: 10.3389/fmicb.2020.583995 (PMC7596291; doi:10.3389/fmicb.2020.583995)
Supplement: Supplementary Figure 1 — Relative abundance of bacterial class among three group samples. PM, swine manure samples; AC, aerobic composting samples; AD, anaerobic digestion samples. [file Data_Sheet_1.docx]

**Fig. S1**| **Relative abundance of bacterial class among three group samples.** PM: swine manure samples, AC: aerobic composting samples, AD: anaerobic digestion samples.


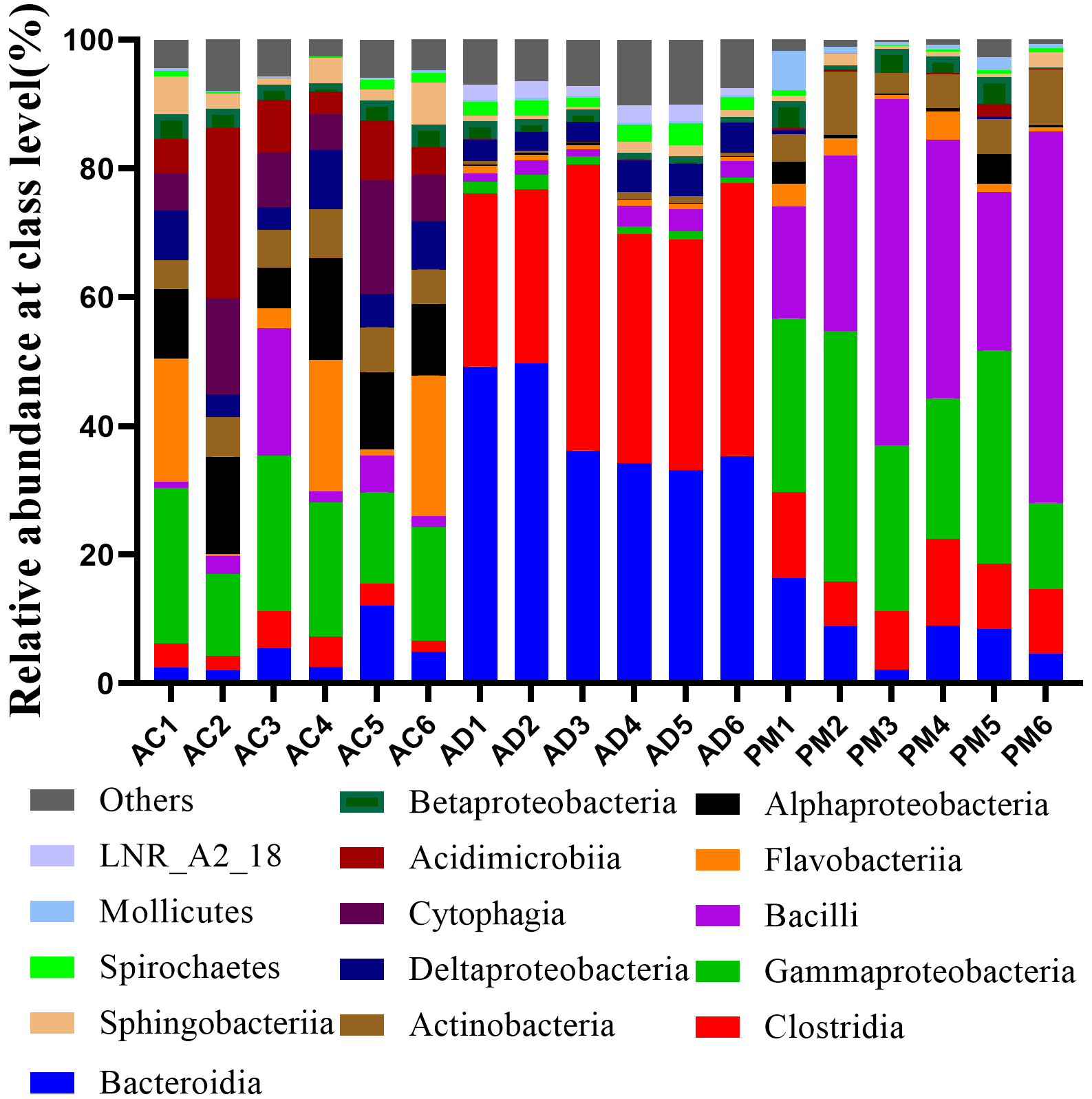


**Fig. S2**| **Comparison of taxonomic distribution of major genera with significant differences (relative abundance of top 30 genus) between two group samples.** PM: swine manure samples, AC: aerobic composting samples, AD: anaerobic digestion samples.


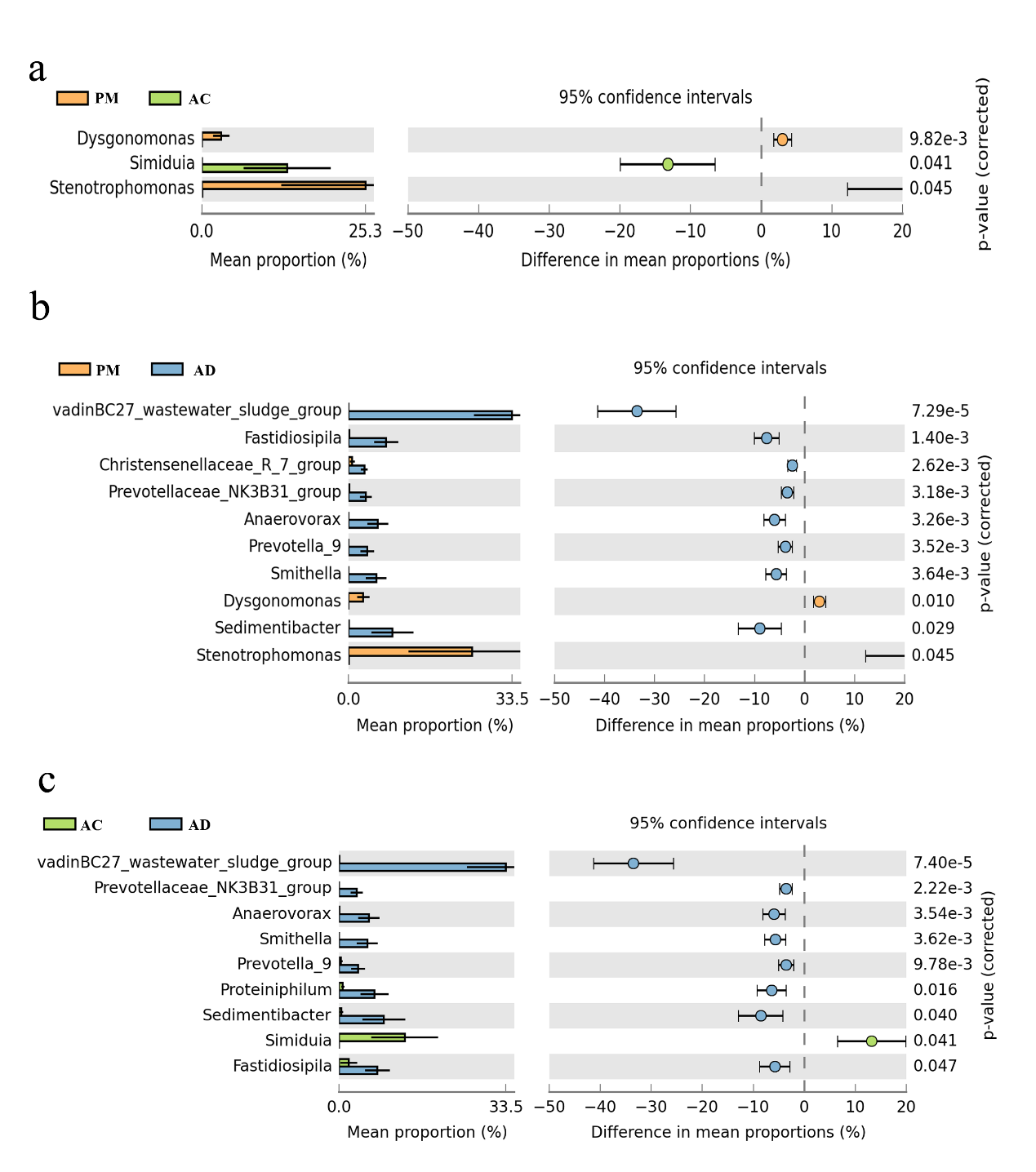


**Fig. S3**| **Phylogenetic molecular ecological networks (pMENs) of bacterial communities at the PM(a), AC(b) and AD(c) groups.** Modules with > 5 nodes were obtained for three group (PM, AC and AD), respectively. The links between two nodes show the correlation (red: positive, blue: negative). The size of cycle indicates the relative abundance of the OTU. The number in the center of cicle represents the modules to which these OTUs belongs. PM: swine manure samples, AC: aerobic composting samples, AD: anaerobic digestion samples.


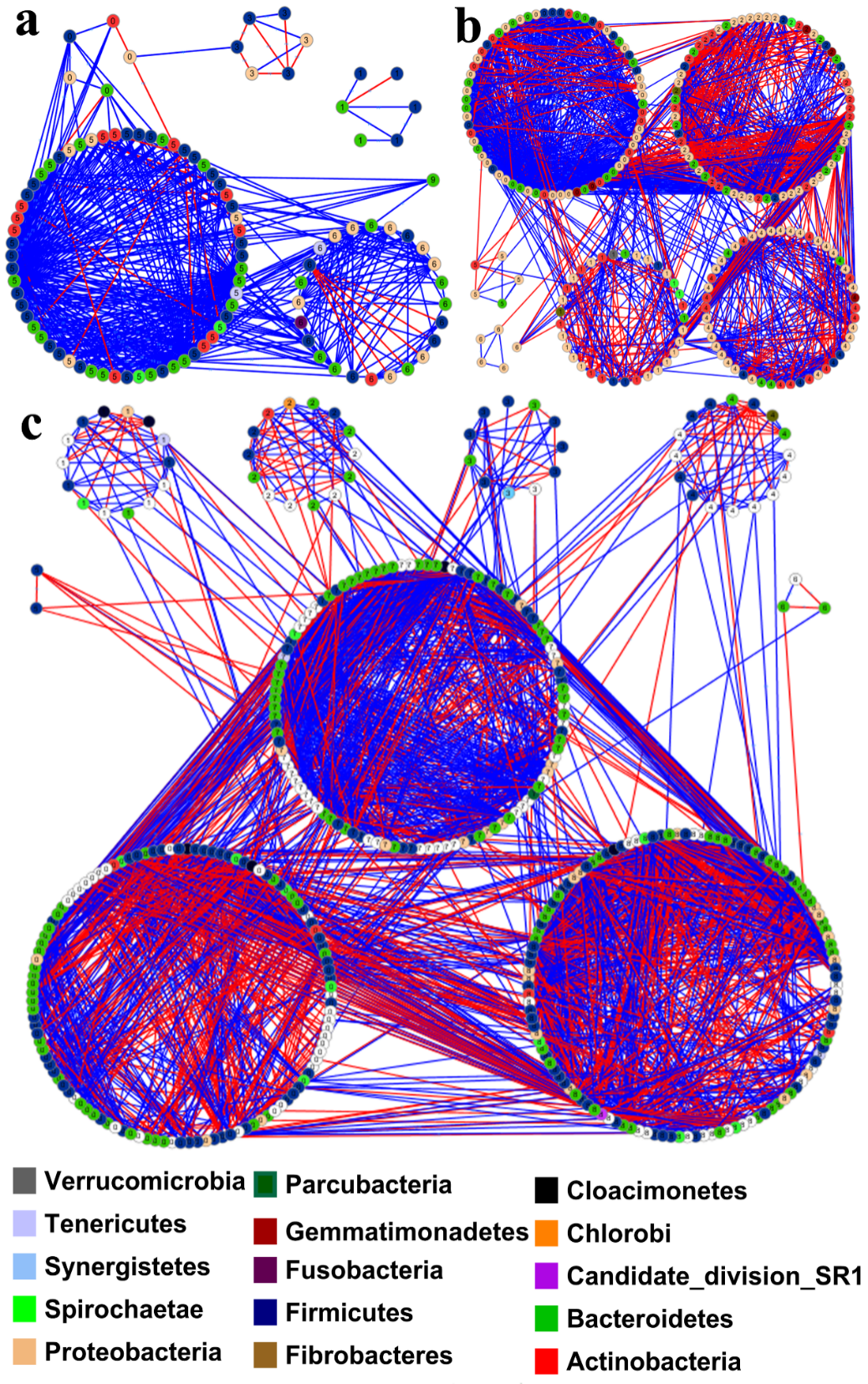


**Fig. S4**| **Summary of module hubs and connectors of the bacterial communities of three groups (PM, AC and AD).** The OTUs were peripherals whose links mainly stayed within their respective modules. Generalists including module hubs (nodes that highly connected with nodes within their modules, Zi > 2.5) and connectors (nodes that connected with several modules, Pi > 0.62). PM: swine manure samples, AC: aerobic composting samples, AD: anaerobic digestion samples.


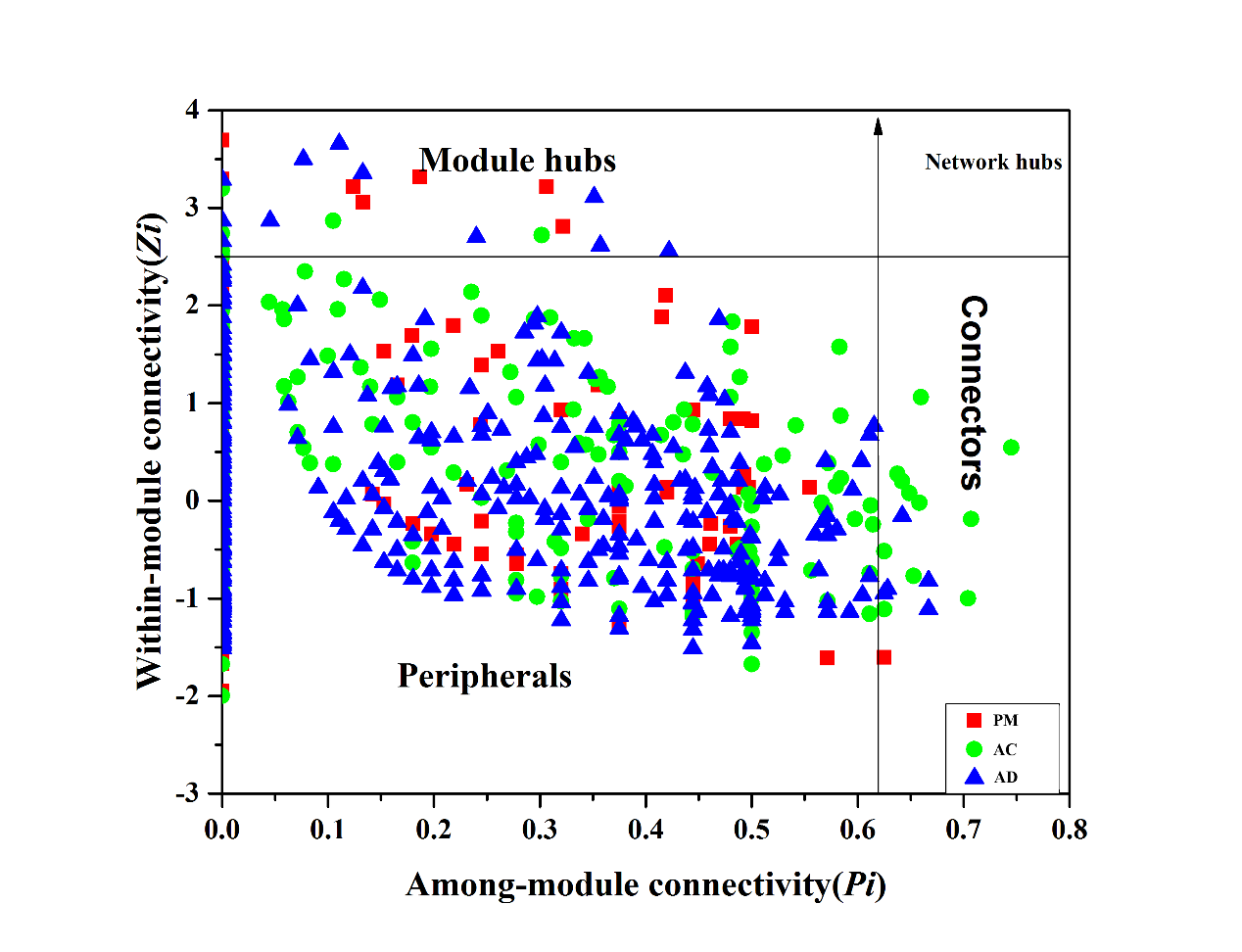


**Table S1 The qPCR primers used in this study.**

| Gene  name | Forward primer | Reverse primer | Annealing temperature |
| --- | --- | --- | --- |
| *tetA* | GCGCGATCTGGTTCACTCG | AGTCGACAGYRGCGCCGGC | 54°C (Aminov et al., 2002) |
| *tetB* | AAAACTTATTATATTATAGTC | TGGAGTATCAATAATATTCAC | 46°C (Aminov et al., 2001) |
| *tetC* | GCGGGATATCGTCCATTCCG | GCGTAGAGGATCCACAGGACG | 59°C (Aminov et al., 2002) |
| *tetE* | GTTATTACGGGAGTTTGTTGG | AATACAACACCCACACTACGC | 54°C (Aminov et al., 2002) |
| *tetG* | GCAGAGCAGGTCGCTGG | CCYGCAAGAGAAGCCAGAAG | 54°C (Aminov et al., 2002) |
| *tetM* | ACAGAAAGCTTATTATATAAC | TGGCGTGTCTATGATGTTCAC | 52°C (Aminov et al., 2001) |
| *tetO* | ATGTGGATACTACAACGCATGAGATT | TGCCTCCACATGATATTTTTCCT | 55°C (Aminov et al., 2001) |
| *tetQ* | AGAATCTGCTGTTTGCCAGTG | CGGAGTGTCAATGATATTGCA | 56°C (Aminov et al., 2001) |
| *tetT* | AAGGTTTATTATATAAAAGTG | AGGTGTATCTATGATATTTAC | 46°C (Aminov et al., 2001) |
| *tetW* | GAGAGCCTGCTATATGCCAGC | GGGCGTATCCACAATGTTAAC | 56°C (Aminov et al., 2001) |
| *tetX* | CAATAATTGGTGGTGGACCC | TTCTTACCTTGGACATCCCG | 55°C (Ng et al., 2001) |
| 16SrRNA | CCTACGGGAGGCAGCAG | ATTACCGCGGCTGCTGG | 60°C (Aminov et al., 2002) |

**Table S2 Physicochemical properties of three group samples.** The difference in physicochemical properties among three group samples was assessed by performing a one-way ANOVA followed by Duncan's multiple range test (p < 0.05). PM: untreated swine manure samples, AC: aerobic composting samples, AD: anaerobic digestion samples.

| Groups | pH | OM | TP | TK | TN |  |
| --- | --- | --- | --- | --- | --- | --- |
| PM | 7.25±0.12a | 782.5±7.78a | 283.5±12.98a | 94±6.63a | 232.67±7.23c | |
| AC | 6.02±0.17b | 396.67±8.43b | 307.83±11.8a | 104.17±6.73a | 508.67±14.08b | |
| AD | 5.33±0.07c | 270.5±8.96c | 305±11.31a | 91.67±8.21a | 887.67±24.82a | |

**Table S3 Spearman’s correlation coefﬁcients between relative abudance of ARGs gene, physicochemical properties, the dominant bacterial populations, and TCs concentration.** ^*^ indicate signiﬁcant value at p < 0.05. ^**^ indicate signiﬁcant value at p < 0.01.

|  | ***tetO*** | ***tetW*** | ***tetQ*** |
| --- | --- | --- | --- |
| **Actinobacteria** | 0.567^*^ | 0.604^**^ | 0.583^*^ |
| **Bacteroidetes** | -0.529^*^ | -0.098 | -0.247 |
| **Cloacimonetes** | -0.748^**^ | -0.713^**^ | -0.652^**^ |
| **Firmicutes** | 0.143 | -0.084 | 0.053 |
| **Gemmatimonadetes** | 0.414 | 0.604^**^ | 0.462 |
| **Proteobacteria** | 0.441 | 0.744^**^ | 0.548^*^ |
| **Spirochaetae** | -0.510^*^ | 0-.442 | -0.254 |
| **Tenericutes** | 0.501^*^ | 0.426 | 0.564^*^ |
| **pH** | 0.766^**^ | 0.624^**^ | 0.849^**^ |
| **OM** | 0.801^**^ | 0.537^*^ | 0.716^**^ |
| **TP** | -0.346 | 0.208 | -0.260 |
| **TK** | 0.317 | 0.133 | 0.234 |
| **TN** | -0.731^**^ | -0.648^**^ | -0.807^**^ |
| ***tetO*** | 1.000 | 0.614^**^ | 0.792^**^ |
| ***tetW*** | 0.614^**^ | 1.000 | 0.707^**^ |
| ***tetQ*** | 0.792^**^ | 0.707^**^ | 1.000 |
| **TC** | 0.684^***^ | 0.761^***^ | 0.783^***^ |
| **OTC** | 0.692^**^ | 0.521^*^ | 0.593^*^ |
| **CTC** | 0.74^***^ | 0.742^***^ | 0.8^***^ |
| **DOC** | 0.796^***^ | 0.769^***^ | 0.777^***^ |

**Table S4 | Mantel analysis on the relationship between the relative abundance of OTUs and environmental factors.** P-values were calculated using the distribution of the Mantel test statistics estimated from 9999 permutations.

| **Physicochemical properties** | **Bray–Curtis** | | **Jaccard** | |
| --- | --- | --- | --- | --- |
|  | **r** | **p** | **r** | **p** |
| **Actinobacteria** | 0.4015 | 0.001 | 0.4135 | 0.001 |
| **Bacteroidetes** | 0.4077 | 0.001 | 0.3462 | 0.001 |
| **Cloacimonetes** | 0.2789 | 0.001 | 0.2270 | 0.022 |
| **Firmicutes** | 0.2264 | 0.006 | 0.2423 | 0.005 |
| **Gemmatimonadetes** | 0.5725 | 0.001 | 0.7054 | 0.001 |
| **Proteobacteria** | 0.6211 | 0.001 | 0.6466 | 0.001 |
| **Spirochaetae** | 0.2071 | 0.022 | 0.1518 | 0.051 |
| **Tenericutes** | 0.0266 | 0.394 | -0.0115 | 0.525 |
| **pH** | 0.5109 | 0.001 | 0.3947 | 0.002 |
| **OM** | 0.5930 | 0.001 | 0.4954 | 0.003 |
| **TP** | -0.0288 | 0.603 | -0.0152 | 0.494 |
| **TK** | -0.0027 | 0.445 | 0.0262 | 0.311 |
| **TN** | 0.6310 | 0.001 | 0.5415 | 0.001 |
| **TC** | 0.502 | 0.002 | 0.4147 | 0.003 |
| **OTC** | 0.4787 | 0.001 | 0.3951 | 0.004 |
| **CTC** | 0.4988 | 0.001 | 0.4133 | 0.003 |
| **DOC** | 0.5619 | 0.001 | 0.4615 | 0.002 |
